# A New Instrument for Extension and Counter-Extension in Injuries of the Lower Extremities

**Published:** 1881-08

**Authors:** 


### Editorial.

### A NEW INSTRUMENT FOR EXTENSION AND COUNTER-EXTENSION IN INJURIES OF THE LOWER EXTREMITIES.

Dr. A. Clendnen, of Fort Lee, N. J., has introduced into practice a new splint for extension and counter extension in injuries of the lower extremities. An account of the instrument is published in the *Medical Gazette* of July 1881, and we are of opinion that it will prove a valuable addition in the treatment of those injuries for which it is intended.

